# American Society of Dental Surgeons

**Published:** 1854-10

**Authors:** Wm. H. Goddard, Chas. Bonsall


### AMERICAN SOCIETY OF DENTAL SURGEONS.

A number of Dentists from different places, who had come on to meet the American Society, having met at Cincinnati on Tuesday, August 1st, 1854, a meeting was held at the Burnet House during the afternoon; present, Drs. W. H. Goddard and Sam'l Griffith of Louisville, Ky., C. W Spalding of St. Louis, Mo., ——Wheeler of Murfreesburg, Tenn., W. H. Dwinelle of Cazanovia, N. Y., H. R. Smith of Terre Haute, Ind., and James Taylor, Charles Bonsall, A. Berry and Joseph Taylor of Cincinnati, O.

Most of the afternoon was passed in an examination of samples of crystal gold, manufactured by Watts & Co., of

Utica, N. Y., and a comparison of plugs made from it and other makes of crystal and foil gold, which examination was conducted partly by the use of a powerful microscope and appeared to satisfy all present of its great superiority over other crystal, and for many cavities, over foil gold.

Before adjournment, the company visited the new building, now in course of completion, for the use of and belonging to the Ohio Dental College Association. The gentlemen present expressed themselves much pleased with the appearance and adaptation of the building to the purposes it is intended for, and considered it highly creditable to the West; particularly as being the only complete College building *belonging to the profession and devoted entirely to the purposes of Dental education.* Adjourned to meet at the house of Dr. James Taylor, at 7, P. M.

Met according to adjournment, when Dr. Goddard was chosen Chairman, and Dr. Bonsall Secretary. On motion the Chair named Drs. Spalding, James Taylor and Smith, a committee on business, who reported a resolution of regret that the notice of the postponement of the meeting of the American Society of Dental Surgeons, to have been held here this day, had not been more generally sent out, particularly to the members of the society, two or three of those present having received no notice; also regretting an apparent misapprehension in relation to the health of the country, together with an assurance that Cincinnati *is, and has been* during the season, almost entirely free from cholera, probably more so than any of the larger Eastern cities; and so far as the necessity for using the *Western Waters* as a medium for reaching Cincinnati is concerned, we would assure our brethren that our country is well supplied with railroads, so that the use of the rivers may be dispensed with for that purpose. The committee suggest the fourth Tuesday, being the 24th day of October, as a proper time for meeting of the society, which would give time for those gentlemen who are connected with any of the Dental schools, to get home before the terms commence on the

6th of November; and we will hope for a full delegation from the North and East, as well as the South and West, at that time, and that we will cordially extend the hospitalities of the West to such of our professional brethren as may then attend.

The foregoing resolutions were approved and after a vote of thanks to our host, adjourned *sine die*.

WM. H. GODDARD, Chairman.

CHAS. BONSALL, Secretary.

